# A Case of Scirrhus of the Thyroid Gland

**Published:** 1844-09-07

**Authors:** R. W. Brown

**Affiliations:** Surgeon to the Bath United Hospital


A CASE OF SCIRRHUS OF THE THYROID GLAND.

By R. W. BROWN, ESQ., Surgeon to the Bath United Hospital.

The case was considered by the author to be interesting in connection with the paper communicated to the Society by Mr. Cæsar Hawkins, on Carcinoma of the Thyroid Gland. The patient, aged 60, began, about Christmas, 1842, to have pain in the larynx, accompanied by hoarseness; and his voice gradually became feeble and stridulous, so that he could scarcely speak above a whisper. A hard swelling soon presented itself in the situation of the left lobe of the thyroid gland, and which could be traced in the direction of the œsophagus. The integument in this part of the neck became thickly studded with hard tubercles of a cancerous character. The patient at length had great difficulty of swallowing, which increased, and he died, after much suffering in June, 1843. On the post-mortem examination, the left lobe of the thyroid gland was found to be the principal seat of disease. It was enlarged, and converted into a mass of carcinomatous substance, white, hard as cartilage, and with some gritty particles dispersed through it. The lymphatic glands on both sides were also converted into the same morbid structure, and compressed the œsophagus. Tumours of the same kind were found in other parts of the body, particularly in the lungs and the liver.—*Trans. Royal Med. and Chirurg. Soc. in Lon. Med. Gaz.*

